# Case Report: Challenges in the etiology of left ventricular aneurysm

**DOI:** 10.12688/f1000research.139038.1

**Published:** 2023-11-20

**Authors:** El Mehdi Channan, Georgiana Pintea Bentea, Brahim Berdaoui, Nasroolla Damry, Marielle Morissens, José Castro Rodriguez

**Affiliations:** 1 Cardiology Department, Brugmann University Hospital, Université Libre de Bruxelles, Brussels, Belgium

**Keywords:** Ventricular aneurysm, Left ventricular non-compaction cardiomyopathy

## Abstract

Left ventricular aneurysms are outpouchings delineated by a thin myocardial wall, more frequently encountered at the apex of the left ventricle, which is seldom dyskinetic or akinetic. Apart from coronary artery disease, the etiology can be challenging. We report the case of a 30-year-old man with an isolated apical left ventricular aneurysm associated with prominent trabeculations on echocardiography.

## Background

Left ventricular aneurysms are outpouchings delineated by a thin myocardial wall, more frequently encountered at the apex of the left ventricle, which is seldom dyskinetic or akinetic ^1^. Apart from coronary artery disease, the etiology can be challenging. We report the case of a 30-year-old man with an isolated apical left ventricular (LV) aneurysm associated with prominent trabeculations on echocardiography.

## Case report

A 30-year-old male, presented to CHU Brugmann in October 2021 with complaints of dyspnea, chest pain, palpitations, and fatigue persisting since January 2021. The patient was born in Armenia and had arrived in Brussels in 2016. He had a medical history of undocumented arrhythmia and systemic tuberculosis at 18 years of age in Armenia. In January 2020, he presented at another hospital with a spontaneous pneumothorax that required hospitalization. He had been smoking since the age of 17 years. Due to his medical condition, he is currently unemployed. His family history included his father’s sudden cardiac death at the age of 55 years.

**Figure 1.**
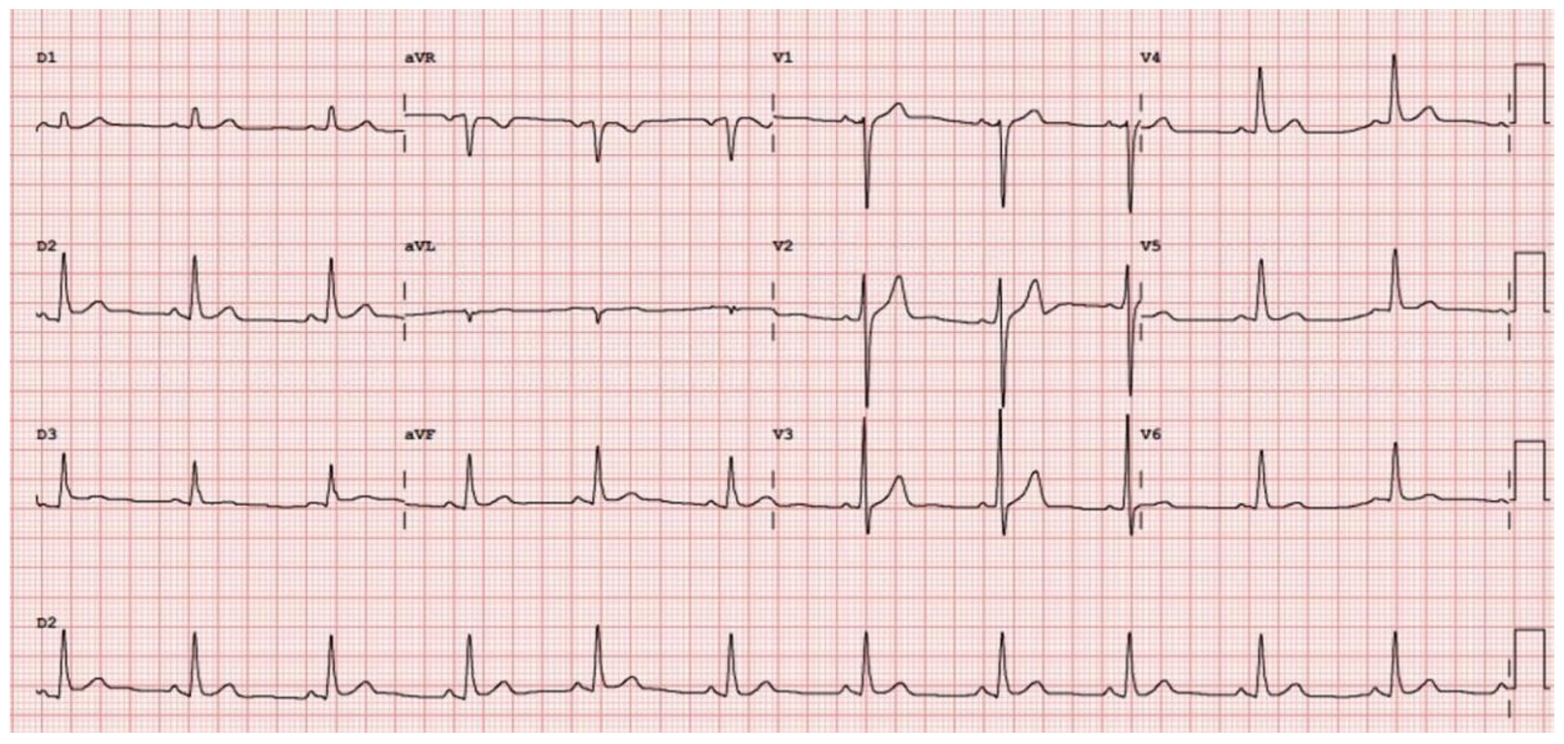
ECG at the initial visit.

**Figure 2.**
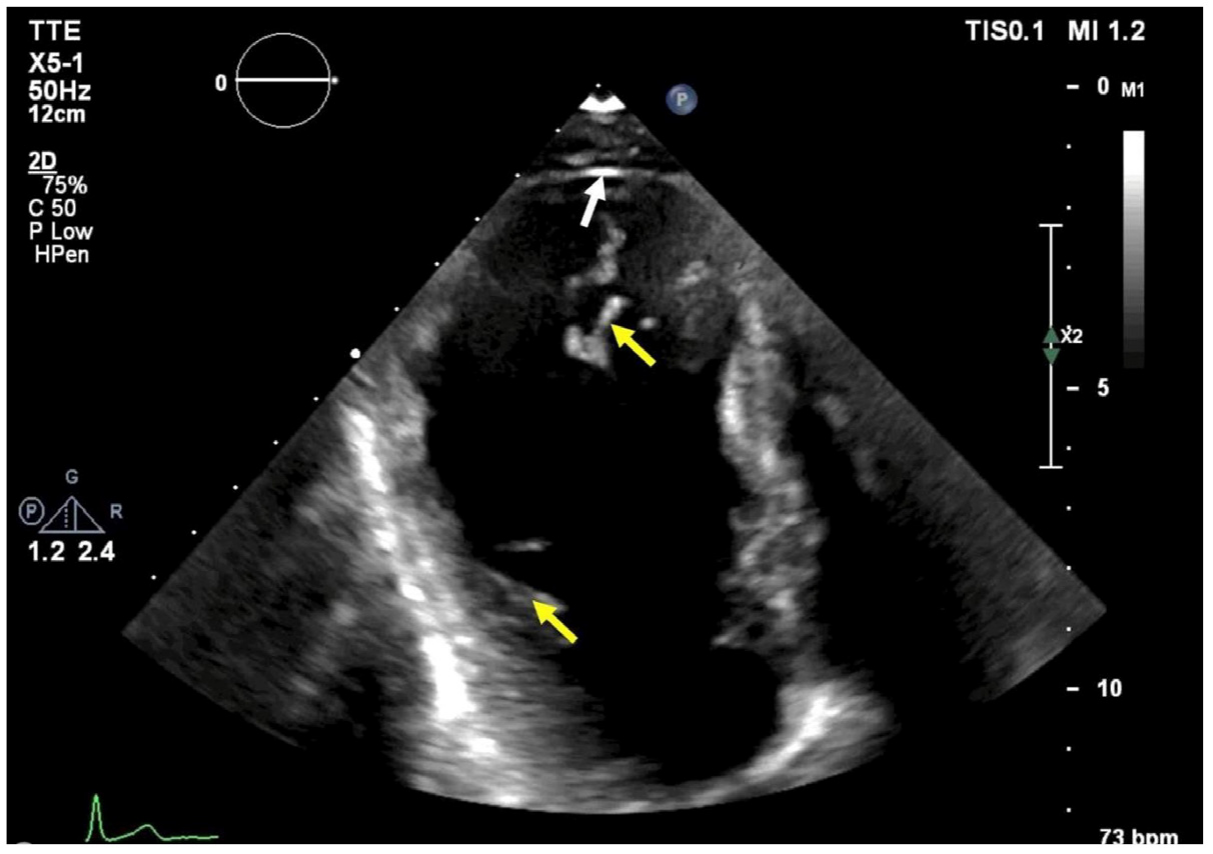
Transthoracic echocardiography demonstrates an isolated apical LV aneurysm (white arrow) associated with trabeculations (yellow arrows).

Electrocardiography (ECG) showed no abnormalities ( Figure 1). Echocardiography revealed moderately decreased left ventricular ejection fraction (LVEF) and isolated apical left ventricular (LV) outpouching associated with marked trabeculations ( Figure 2).

**Figure 3.**
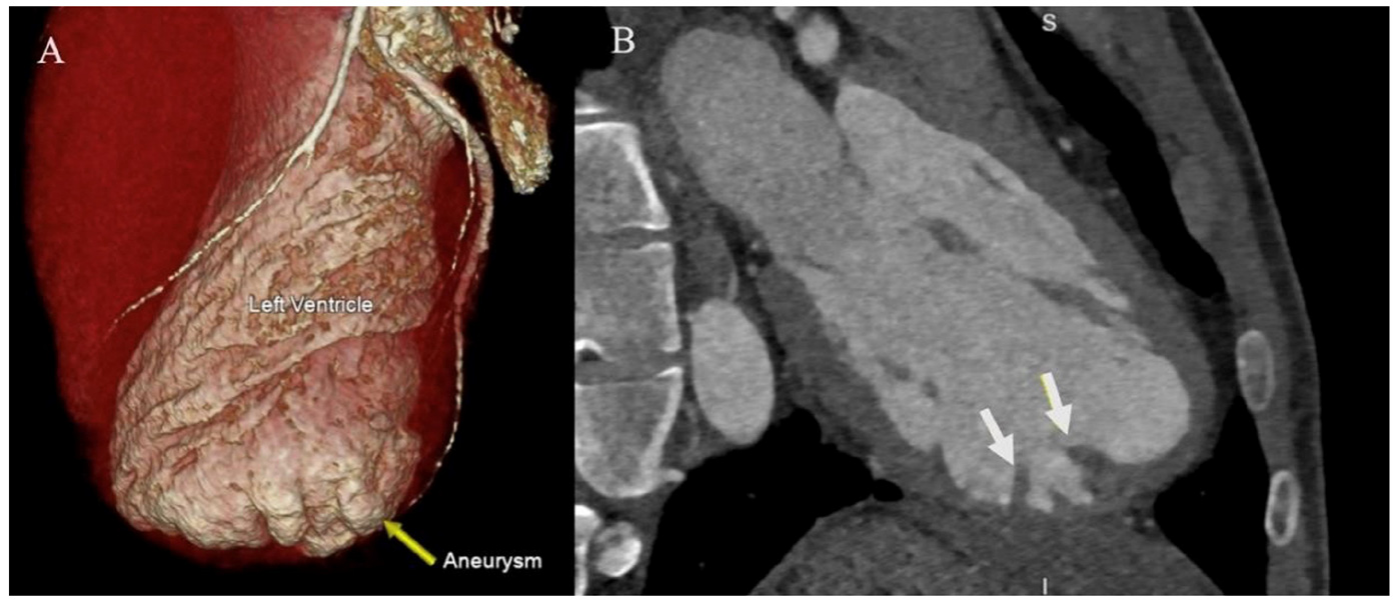
Cardiac CT imaging revealed an isolated apical LV aneurysm (yellow arrow) with apical trabeculations (white arrows).

**Figure 4.**
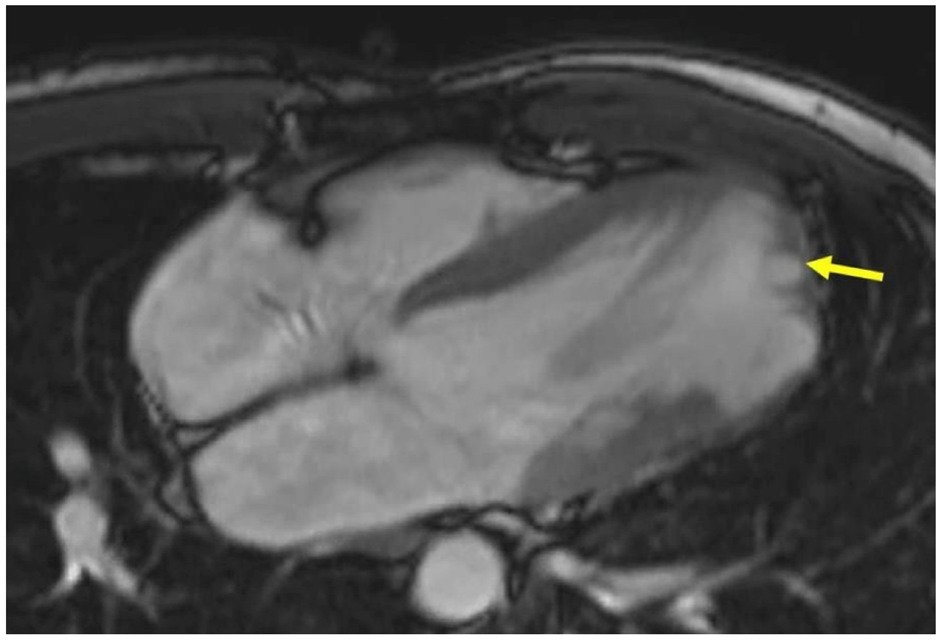
CMR revealed an isolated apical LV aneurysm (yellow).

A coronary angiogram was performed, which revealed normal coronary arteries. A cardiac computed tomography (CT) scan and magnetic resonance (CMR) confirmed that the apical outpouching was indeed a LV aneurysm ( Figure 3 and Figure 4).

An implantable loop recorder revealed one episode of non-sustained ventricular tachycardia. An electrophysiological study was performed without inducing either ventricular or supraventricular arrythmia.

Serological screening for *Trypanosoma cruzi* was negative. Furthermore, there was no α-1-antitrypsin enzyme deficiency. The patient had no skeletal, vascular, or ophthalmologic signs suggestive of connective tissue disease.

We concluded that our patient has a LV apical aneurysm with a normal coronary artery. After one year of follow up his condition did not improve even with the introduction of an ACE inhibitor for mildly reduced ejection fraction.

## Discussion

The patient’s echocardiography revealed an apical outpouching consistent with a myocardial aneurysm or pseudoaneurysm. Differentiation between these two entities can be challenging. An aneurysm is an outpouching containing a thin myocardial layer. Pseudoaneurysm represents a pericardial-covered cardiac rupture, as such; it does not include a myocardial layer ^1^. Cardiac CT scan and CMR helped us establish the diagnosis of an apical aneurysm by visualizing a thin myocardial layer.

There are ischemic and non-ischemic causes of apical aneurysm. The most common cause of apical aneurysm is myocardial infarction ^1^. Coronary angiography showed normal coronary arteries, and CMR showed no signs suggestive of ischemic myocardial disease. Regarding the non-ischemic causes, α-1-antitrypsin enzyme deficiency is associated with pulmonary emphysema, and spontaneous pneumothorax and, in rare cases, may be associated with ventricular pseudoaneurysm ^2^. The patient’s chest CT showed severe emphysema for his age, and he had a spontaneous pneumothorax a year earlier.

Therefore, the level of α-1-antitrypsin enzyme was determined and was within the normal range. The patient underwent a comprehensive autoimmune and infectious serology assessment. We found no evidence of an autoimmune disease (negative anti-nuclear antibody and anti-neutrophil cytoplasmic antibody) or evidence of connective tissue disease (normal ophthalmologic and physical examination, an aortic root within normal range on echocardiography, and normal thoracic aorta on the CT-scan). Infectious serology screening, including serology for *Trypanosoma cruzi,* was negative. As a reminder, the patient had a history of invasive systemic tuberculosis, and the aneurysm may be consistent with a mycotic aneurysm as a complication of systemic tuberculosis ^3^. However, the CMR did not showed any arguments in favor of a left ventricular aneurysm secondary to a myocarditis, such as gadolinium late enhancement. Another hypothesis for the etiology could be a congenital LV aneurysm, which contrasts with the familial history of sudden death.

**Figure 5.**
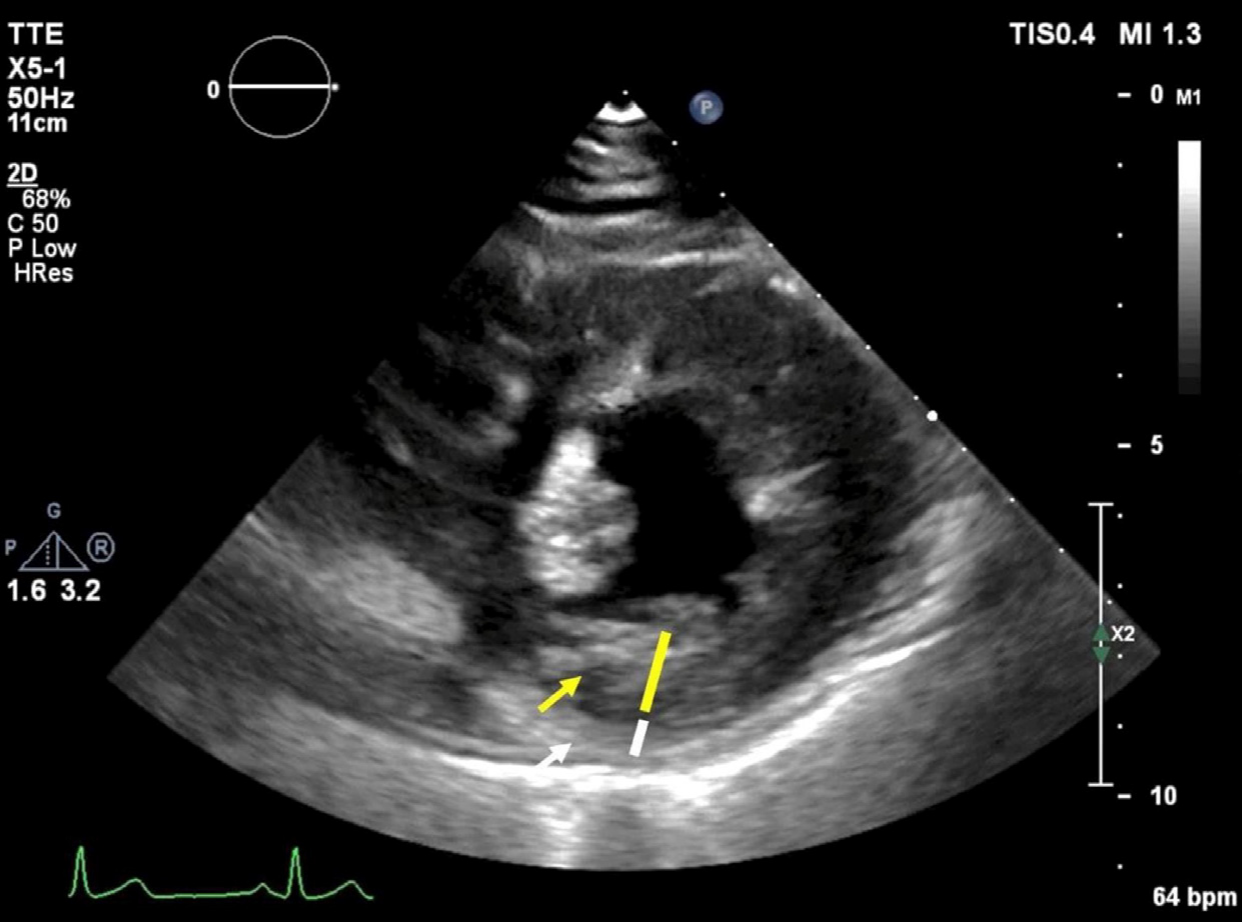
Transthoracic echocardiography demonstrates a ratio of the systolic thickness of the noncompacted (yellow line) to compacted layer (white line) greater than two in the parasternal short axis view.

**Figure 6.**
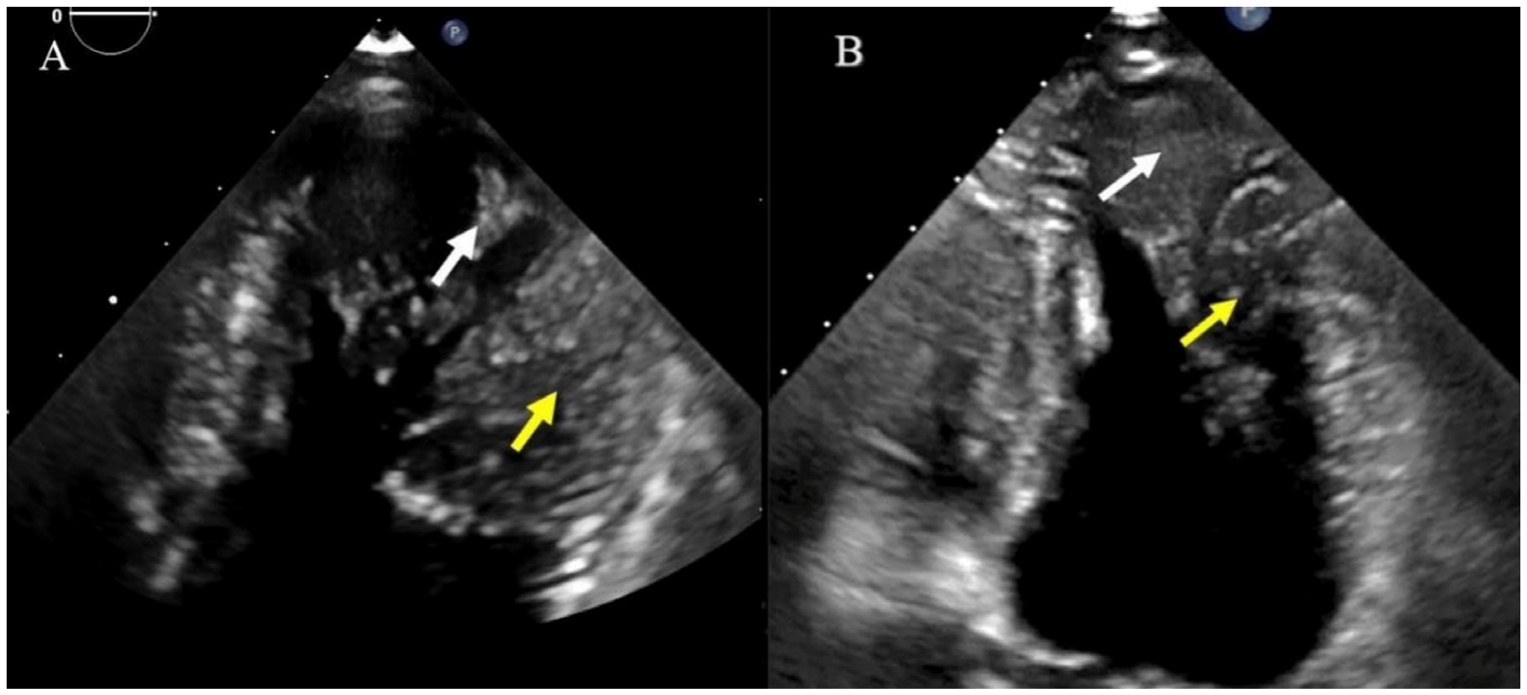
Distribution of the trabeculations at the level of the LV apical aneurysm (white arrow) and the mid-lateral segment (yellow arrow).

**Figure 7.**
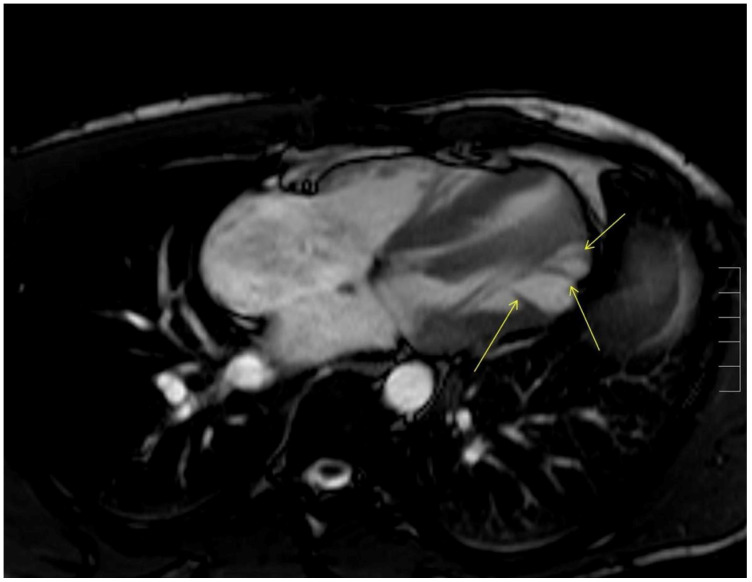
CMR show apical and mid-lateral distribution of the trabeculations (yellow arrow).

However, after reviewing the imaging studies, we found that the patient had marked prominent myocardial trabeculations localized at the level of the LV aneurysm, and met the Jenni criteria ^4^ for left ventricular non-compaction cardiomyopathy (LVNC): 1) thickened myocardium with a noncompacted inner layer and a compacted outer layer ( Figure 5), 2) the ratio of the systolic thickness of the noncompacted to the compacted layer is greater than two in the parasternal short-axis view ( Figure 5), 3) color Doppler evidence of deep intertrabecular recesses filled with blood from the LV cavity, 4) the typical distribution of affected segments includes the apical LV and the mid-lateral segment, as in our patient. ( Figure 6). These findings were confirmed by Petersen criteria in CMR ^5^ ( Figure 7). The family history of his father's sudden cardiac death supports this hypothesis. The results of genetic testing excluded collagen defects and showed no mutation suggestive of LVNC, but as we know, genetic testing has a low diagnostic yield for LVNC ^6^.

Treatment of heart failure with a mildly reduced ejection fraction was initiated with an ACE inhibitor, a beta blocker, and a SGLT-2 inhibitor, resulting in improvements of LVEF and symptoms. Defibrillator implantation was discussed but not yet performed, because the patient does not currently meet the criteria for a defibrillator. The patient has understood his illness well and has been compliant with all the investigations. However, currently, he does not see any significant improvement in his condition.

In conclusion, this young patient presents a LV apical aneurysm associated with a limited form of LVNC. This is a rare association, and to our knowledge, there are few similar cases in the literature to date. The diagnosis may be made in newborns ^7^, in children ^8^, in adults as in our case, or in older adults. This unusual combination of findings makes the case valuable for medical literature, as it adds to the understanding of diverse cardiac pathologies. The report demonstrates a thoughtful approach to ruling out potential causes of left ventricular aneurysm by considering both ischemic and non-ischemic etiologies. The inclusion of serological and genetic testing helps exclude various conditions, leading to a more accurate diagnosis. However, the case report does not include long-term follow-up data to assess disease progression over time. Although we provide a comprehensive assessment, the exact cause of the left ventricular aneurysm and the association with limited form of LVNC remains uncertain. A larger sample size or case series would be needed to draw more robust conclusions and further support the observed association between the left ventricular aneurysm and limited form of LVNC.

Overall, our case report offers valuable insights into a rare cardiac condition, utilizing multiple diagnostic techniques and considering various potential causes. However, it would benefit from long-term follow-up data and a larger sample size to enhance the generalizability of the findings and explore potential causality in this unique association.

## Consent

Written informed consent for publication of their clinical details and clinical images was obtained from the patient.

## Data Availability

All data underlying the results are available as part of the article and no additional source data are required.
