# Supplementary material for: Case Report: Challenges in the etiology of left ventricular aneurysm
Source: F1000Res. 2023 Nov 20;12:1486. [Version 1] doi: 10.12688/f1000research.139038.1 (PMC11112464; doi:10.12688/f1000research.139038.1)
Supplement: Peer review history

# Reviewer report: Reviewer response for version 1

**DOI:** 10.5256/f1000research.152278.r245312

**Published:** 2024-05-11

**Authors:** Daniela Corina MIRICA

**Affiliations:** 1 Hopital Delta, Auderghem, Bruxelles, Belgium

The manuscript under review presents a medical case with significant academic potential. However, there are several areas requiring attention and improvement.  Below are detailed observations and suggestions for revision.

**Background and Abstract:**

 The background section duplicates the abstract without providing additional context or depth to the research question or the case's significance. A more distinct background section is necessary to set the stage for the case presentation, outlining the current understanding and gaps in knowledge regarding the topic.

**Necessity of Coronary Angiogram:**

 The manuscript lacks a comprehensive explanation of the need for a coronary angiogram in the context of alternative non-invasive imaging modalities, especially for a low-risk patient for coronary artery disease (CAD). It is crucial to justify the choice of invasive diagnostic methods over non-invasive options, considering the patient's risk profile and existing literature on the subject.

**Structure of Case Presentation:**

 The structure of the case presentation requires refinement. It should systematically follow the sequence of events, from the initial presentation through all diagnostic procedures to the final diagnosis, ensuring clarity and logical flow. Each finding from various explorations, such as MRI results and genetic testing, should be detailed within the case report section to provide a comprehensive overview of the patient's condition and the diagnostic journey.

**Discussion on Diagnostic Hypotheses and Differential Diagnosis:**

 The discussion section begins with the main diagnostic hypothesis but lacks a robust argumentative foundation to support it. It should include a detailed analysis of why this hypothesis was considered over others, supported by evidence from the case and relevant literature. Following this, a thorough differential diagnosis should be presented, comparing and contrasting the case with similar reports in the literature to contextualize the findings and the decision-making process.

**Case Report Description:**

 All findings, particularly those from MRI and genetic testing, need to be more comprehensively described within the case report section. This detail will enrich the case's educational value, allowing readers to understand the diagnostic significance of these tests fully.

**Conclusion and Recommendations for Revision:**

 While the case presents an interesting scenario with potential for significant academic contribution, it requires major modifications to enhance its clarity, depth, and educational value. The authors are encouraged to:

1. Distinguish the background from the abstract with additional context.
2. Justify the use of coronary angiogram in a low-risk patient for CAD, especially in the presence of non-invasive alternatives.
3. Reorganize the case presentation for a clearer, chronological depiction of findings and diagnoses.
4. Expand the discussion to include a stronger foundation for the main diagnostic hypothesis and a detailed differential diagnosis.
5. Provide a more detailed description of all findings, especially those from MRI and genetic testing, within the case report section.

Are enough details provided of any physical examination and diagnostic tests, treatment given and outcomes?

Partly

Is the case presented with sufficient detail to be useful for other practitioners?

Yes

Is sufficient discussion included of the importance of the findings and their relevance to future understanding of disease processes, diagnosis or treatment?

Partly

Is the background of the case’s history and progression described in sufficient detail?

Partly

Reviewer Expertise:

cardiology, non-invasive imaging

I confirm that I have read this submission and believe that I have an appropriate level of expertise to confirm that it is of an acceptable scientific standard, however I have significant reservations, as outlined above.

# Reviewer report: Reviewer response for version 1

**DOI:** 10.5256/f1000research.152278.r265728

**Published:** 2024-05-02

**Authors:** Akinori Higaki

**Affiliations:** 1 Ehime University, Toon, Japan

In this report, the authors present a case of left ventricular non-compaction cardiomyopathy (LVNC) with associated apical aneurysms amidst prominent trabeculations. Given the rarity of this combination, their detailed report significantly contributes to the field of medicine. However, the reviewer notes several minor shortcomings that merit improvement.

 Firstly, the authors mention α-1-antitrypsin enzyme deficiency as a cause of non-ischemic etiology of ventricular aneurysms, but there are other common etiologies such as cardiac sarcoidosis and hypertrophic cardiomyopathy. Additionally, MINOCA (myocardial infarction with non-obstructive coronary arteries) can be considered an ischemic etiology of aneurysm. The discussion on etiology could be enriched by referencing previous literature, for instance, Shan et al [Ref 1].

 Regarding the manuscript's structure, it is unusual to include case descriptions in the Discussion section, especially when referencing specific figures like Figures 5, 6, and 7. These descriptions would be more suitable in the case report section. The Discussion section should instead focus on analytical discussions.

 Finally, the reviewer suggests modifying the manuscript title to a more direct expression. For example, consider stating that the case report discusses a left ventricular aneurysm associated with LVNC. This change will help broaden the readership.". The reviewer suggests a more direct approach, such as stating that the case report discusses a left ventricular aneurysm associated with LVNC. The reviewer suggests a more direct approach, such as stating that the case report discusses a left ventricular aneurysm associated with LVNC.

Are enough details provided of any physical examination and diagnostic tests, treatment given and outcomes?

Yes

Is the case presented with sufficient detail to be useful for other practitioners?

Partly

Is sufficient discussion included of the importance of the findings and their relevance to future understanding of disease processes, diagnosis or treatment?

Partly

Is the background of the case’s history and progression described in sufficient detail?

Yes

Reviewer Expertise:

Cardiology and cardiovascular disease.

I confirm that I have read this submission and believe that I have an appropriate level of expertise to confirm that it is of an acceptable scientific standard, however I have significant reservations, as outlined above.

## References

[1] : Coexistence of congenital left ventricular aneurysm and prominent left ventricular trabeculation in a patient with LDB3 mutation: a case report. *J Med Case Rep* .2017;11(1) : 10.1186/s13256-017-1405-1 229 10.1186/s13256-017-1405-1 28821295 PMC5563034

# Reviewer report: Reviewer response for version 1

**DOI:** 10.5256/f1000research.152278.r245309

**Published:** 2024-02-26

**Authors:** Anna Chaloupka

**Affiliations:** 1 Masaryk University, Brno, Czech Republic

Summary

 The article presents a detailed case report of a 30-year-old male with an isolated apical left ventricular (LV) aneurysm associated with prominent trabeculations, identified through echocardiography. The case is notable for its thorough investigation into the etiology of the aneurysm, ruling out common causes such as coronary artery disease and exploring less common factors. The diagnosis of left ventricular non-compaction cardiomyopathy (LVNC) is considered based on imaging findings and the patient's familial history.

 The article is well-organized, with a clear division into sections that logically progress from the patient's background and case report through the discussion of diagnostic findings and conclusions. The detailed account of the patient's symptoms, medical history, and the diagnostic tests performed contributes to a thorough understanding of the case. 

 The methodology, consisting of various diagnostic tests including echocardiography, coronary angiogram, cardiac CT, and CMR, is appropriately chosen for the case's complexity and supports the reproducibility of the diagnostic conclusions. The inclusion of genetic testing, despite its low diagnostic yield for LVNC, adds depth to the investigation. 

 The article cites current literature, particularly in discussing the differentiation between myocardial aneurysm and pseudoaneurysm, the causes of apical aneurysms, and the imaging diagnostic criteria for LVNC. The discussion could be strengthened by including a broader context of how this case fits into the existing literature on LVNC especially in light of the most recent ESC cardiomyopathy guidelines from 2023. There is a compelling argument for discontinuing the use of the term LVNC in favor of "left ventricular hypertrabeculation." This shift reflects a deeper understanding of the condition's nature, acknowledges the complexity of cardiac phenotypes, and aligns with a more evidence-based, nuanced approach to cardiac anomalies. 

 The case is presented with sufficient detail to be useful for teaching or for other practitioners, particularly in illustrating the diagnostic approach to a complex case of LV aneurysm. The detailed description of the diagnostic process, including the rationale for each test and the interpretation of results, provides valuable insights for medical education and clinical practice. However it would be useful to mention the recent critical reassessment of the condition as per the recent guidelines from 2023 which challenge the use of LVNC as a definitive cardiomyopathy diagnosis.

Are enough details provided of any physical examination and diagnostic tests, treatment given and outcomes?

Yes

Is the case presented with sufficient detail to be useful for other practitioners?

Partly

Is sufficient discussion included of the importance of the findings and their relevance to future understanding of disease processes, diagnosis or treatment?

Yes

Is the background of the case’s history and progression described in sufficient detail?

Yes

Reviewer Expertise:

cardiomyopathy

I confirm that I have read this submission and believe that I have an appropriate level of expertise to confirm that it is of an acceptable scientific standard.

## References

[1] : 2023 ESC Guidelines for the management of cardiomyopathies. *Eur Heart J* .2023;44(37) : 10.1093/eurheartj/ehad194 3503-3626 10.1093/eurheartj/ehad194 37622657

# Response

**Published:** 2024-03-03

**Authors:** Dr Synapse

Thank you very much for taking the time to review my publication. Your feedback and insights are greatly appreciated and will be invaluable in improving my work. I am grateful for your expertise and thoughtful commentary. Thank you for contributing to the quality and depth of my research

 Mehdi
